# Supplementary material for: Seminoma and Embryonal Carcinoma Footprints Identified by Analysis of Integrated Genome-Wide Epigenetic and Expression Profiles of Germ Cell Cancer Cell Lines
Source: PLoS One. 2014 Jun 2;9(6):e98330. doi: 10.1371/journal.pone.0098330 (PMC4041891; doi:10.1371/journal.pone.0098330)
Supplement: File S1 — ZIP file containing DMRforPairs output for significant regions. Please start from the html files. (ZIP) [file pone.0098330.s008.zip › figures/20652.pdf]

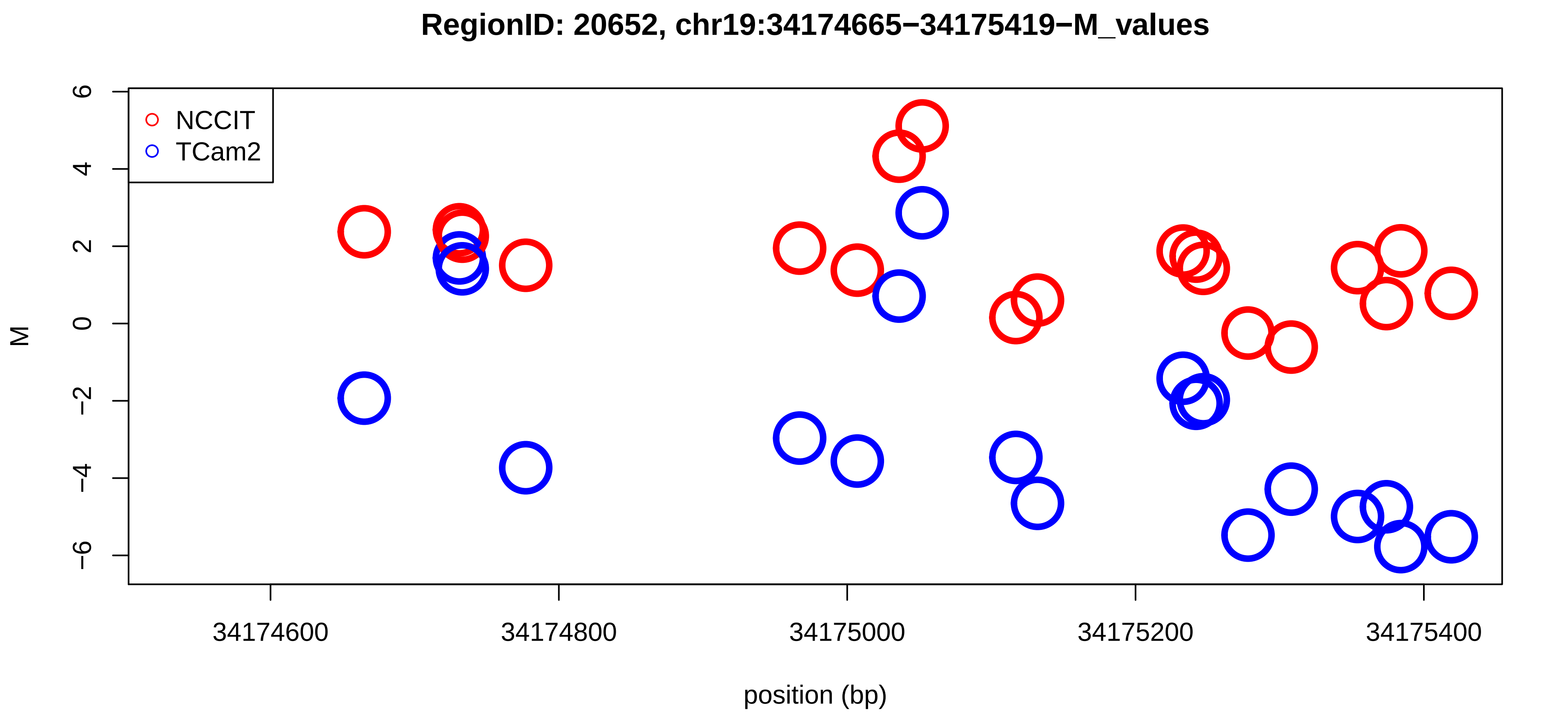

RegionID: 20652, chr19:34174665−34175419−M_values
NCCIT
TCam2
M
position (bp)
34174600
34174800
34175000
34175200
34175400

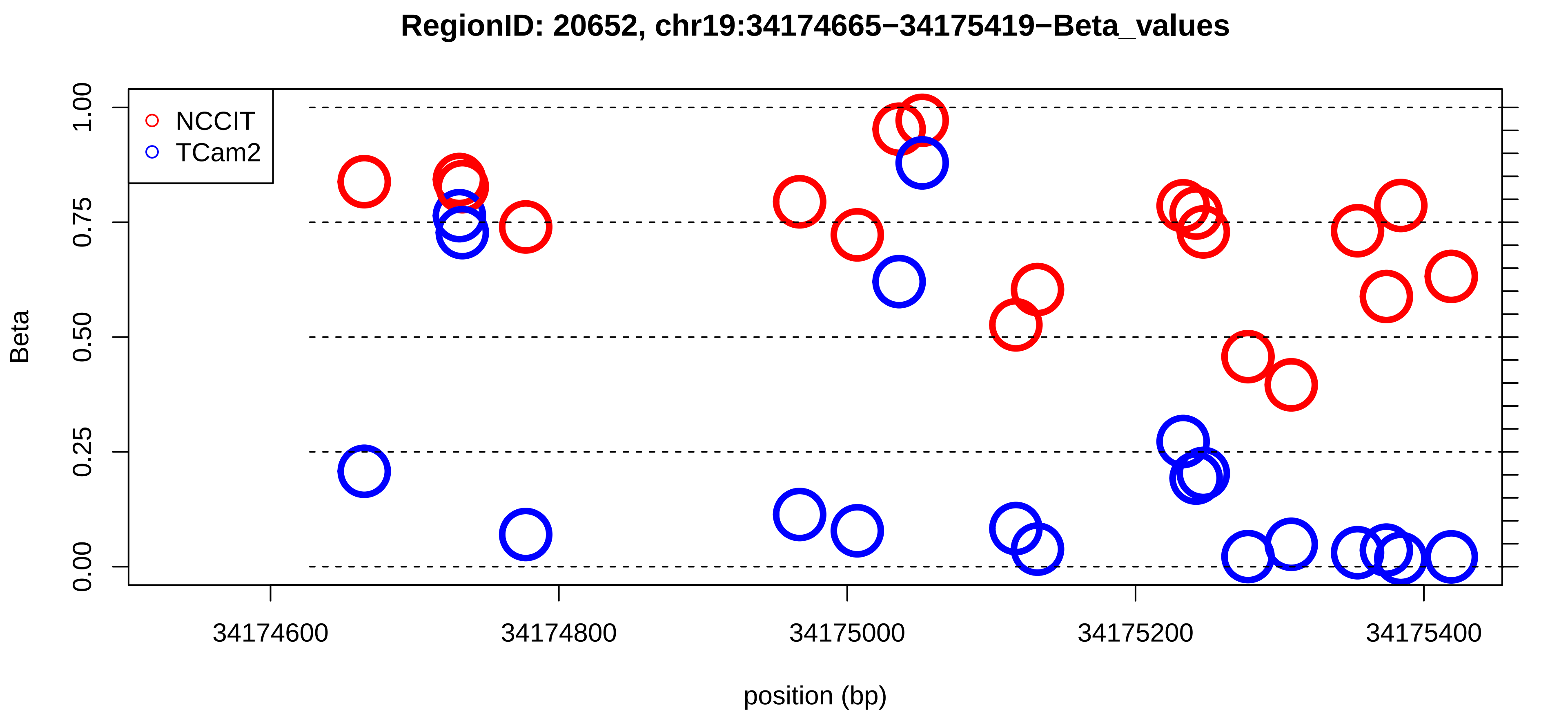

RegionID: 20652, chr19:34174665−34175419−Beta_values
NCCIT
TCam2
Beta
position (bp)
34174600
34174800
34175000
34175200
34175400

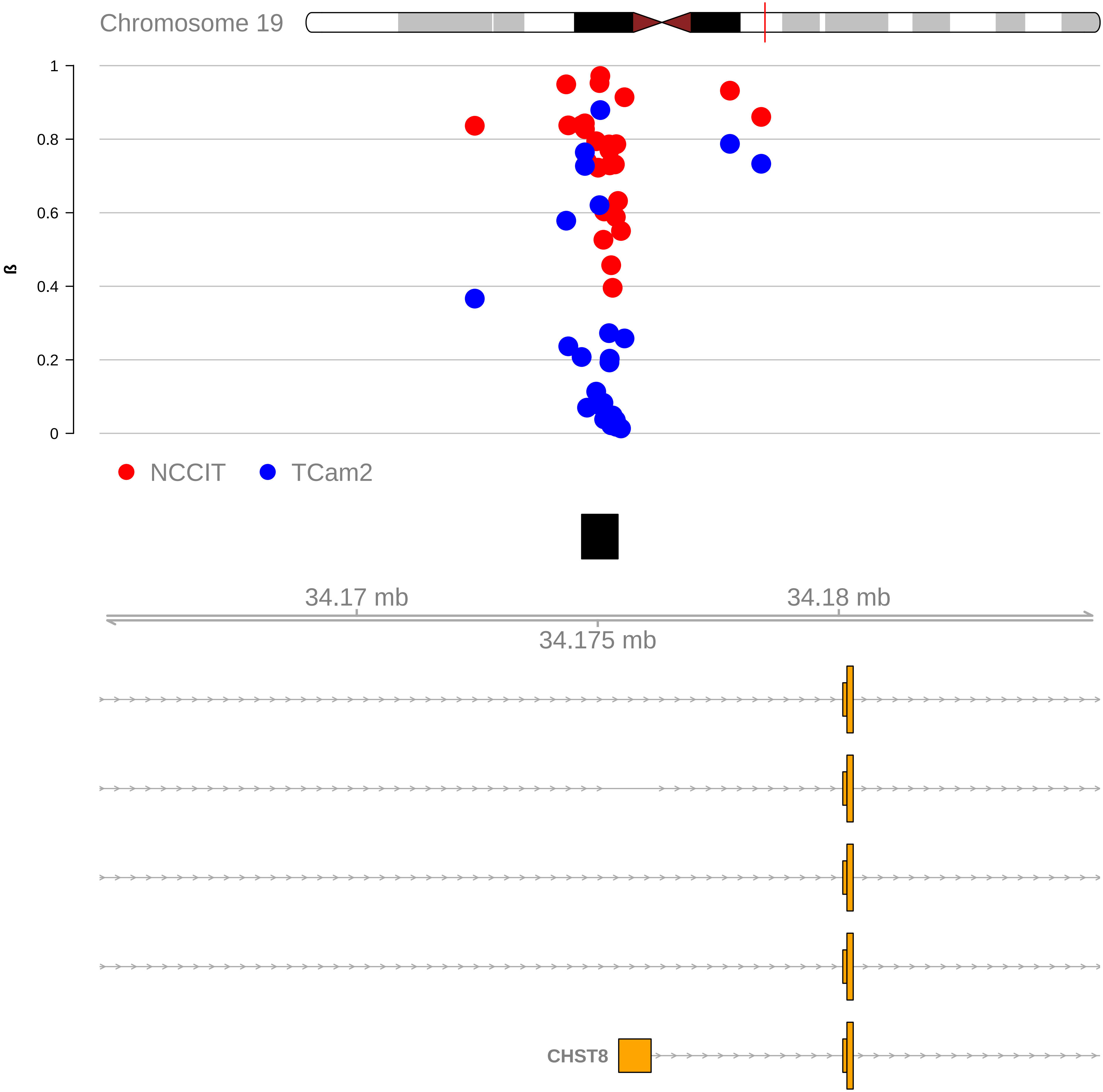

Chromosome 19
1
0.8
0.6
0.4
0.2
0
ß
NCCIT
TCam2
34.17 mb
34.18 mb
34.175 mb
CHST8
